# An Experience in Laboratory Diagnosis of Fungal Infections in COVID -19 Patients

**DOI:** 10.1055/s-0043-1768140

**Published:** 2023-09-26

**Authors:** Sushma Yadav Boorgula, Sadhana Yelamanchili, Srinivas Kishore Sistla, Lubna Saher, Deepika Gujjarlapudi, Shalini E., Sindhu Devi V., Nageshwar Reddy Duvvur

**Affiliations:** 1 Department of Microbiology, AIG Hospitals, Hyderabad, Telangana, India; 2 Department of Otolaryngology, AIG Hospitals, Hyderabad, Telangana, India; 3 Department of Biochemistry, AIG Hopsitals, Hyderabad, Telangana, India; 4 Department of Gastroenterology, AIG Hospitals, Hyderabad, Telangana, India

**Keywords:** COVID-19, sars-cov-2, diabetes mellitus, steroids, fungus

## Abstract

**Introduction** Coronavirus disease 2019 (COVID-19) caused by severe acute respiratory syndrome coronavirus 2 (SARS-CoV-2) has cast a gloom spell on healthcare worldwide, infecting millions of people.

**Objective** The aim of the present study is to determine the prevalence and review the contributing comorbidities and the precipitating factors leading to the emergence of the fungal infections in COVID-19-affected patients. To assess the utility of different laboratory techniques for confirmation of fungal infections. To assess the strengths and limitations of the diagnostic methods.

**Methods** We have studied 252 clinical samples obtained from 121 COVID-positive patients.

**Results** Among the 121 patients clinically diagnosed with fungal infections, 88 had diabetes and were given steroids for treatment ( *p* -value = 0.001). Ninety-five patients (78.5%) had a positive laboratory diagnosis (either culture positive, potassium hydroxide [KOH]-positive or positive histopathology report). Fungal culture was positive in 75 (61.9%) patients and histopathology report was positive in 62 (51.2%). Histopathology was positive in 7 (5.8%) patients in whom culture and KOH were negative.

**Conclusion** Aggressive treatment methods, administration of immune suppressants, and antibiotics, with an intention to salvage, have made patients susceptible to the benign fungus, causing it to evade the host immunity, thus leading to invasive infections. Applying different laboratory modalities would not only aid in providing fast and valuable information but also help in understanding the pathology which would assist the clinician in selecting the correct treatment for the patient.

## Introduction

Coronavirus disease 20191 (COVID-19) caused by severe acute respiratory syndrome corona virus 2 (SARS-CoV-2) has cast a gloom spell on healthcare worldwide, infecting millions of people. The impact on mankind has been significant since then. 1 2 3 4

The epidemic of respiratory infection due to the new coronavirus SARS-CoV-2 that emerged by the end of 2019 in China has become a pandemic and associated has been associated with a huge number of deaths. The mortality largely varies between countries, with some countries having unexplained high rates. With many causes of morbidity and mortality in COVID-19 patients, the frequency and impact of coinfections have still been poorly studied, particularly in patients with an acute respiratory distress syndrome. Patients admitted in intensive care units (ICUs) for COVID-19 share risk factors and underlying diseases reported for invasive fungal infections, particularly chronic respiratory diseases, corticosteroid therapy, intubation/mechanical ventilation, cytokine storm. 5

Most of the patients affected by COVID had a prolonged stay at the hospital, developing co-infections as frequent complications. 6 7 This led to altered human microbiota in patients infected with COVID-19, responsible for secondary infections (coinfections or superinfections) often caused by bacterial and fungal isolates. 8 9 10 A relative higher incidence of varied fungal coinfections were recognized in COVID-19 patients. 11 12 13 Due to laxity in approach toward fungal disease, morbidity and mortality is expected to worsen in covid 19.Therefore it is important to have an efficient treatment of the fungal coinfections, to reduce morbidity and mortality. 14

It is also reported that patients with COVID-19 have increased mortality due to fungal infections, mainly due to impaired immune responses, making it imperative for an effective diagnostic and treatment approach. 15 16

## Aim and Objective

➢ The aim of the present study is to determine the prevalence and review the contributing comorbidities and the precipitating factors leading to the emergence of fungal infections in COVID-19-affected patients.
➢ To assess the utility of different laboratory techniques for confirmation of fungal infections.
➢ To assess the strengths and limitations of the diagnostic methods.

## Materials and Method

**Fig. 1:**
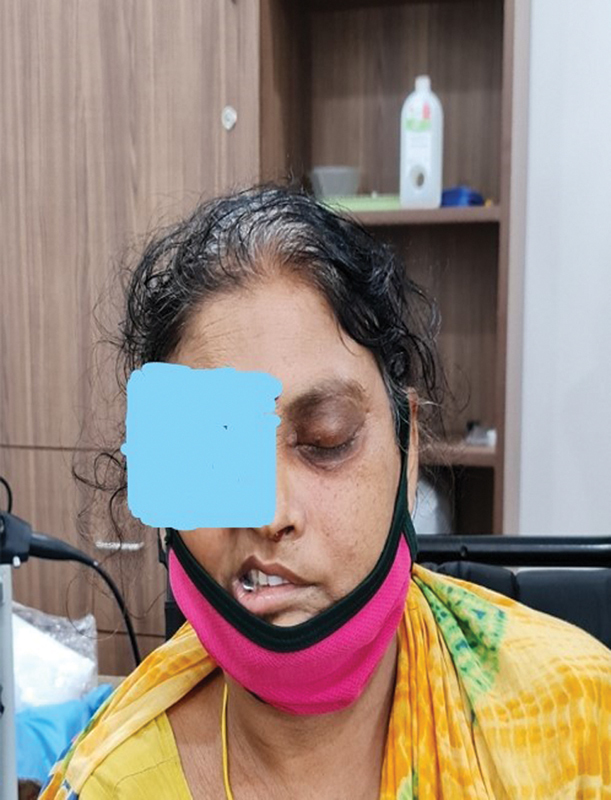
A Middle aged lady with an orbital swelling post Covid.

**Fig. 2:**
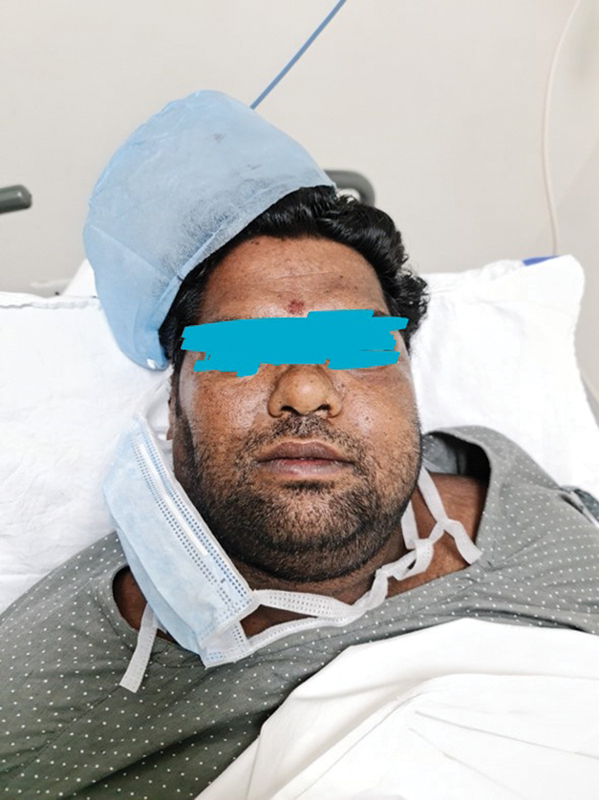
A Gentleman presented with a painful cheek swelling post Covid.

The clinical samples were collected from patients who developed signs and symptoms of fungal infections affecting various organs, and who had been diagnosed with severe acute respiratory syndrome due to COVID-19. ( Figs. 1 and 2 )

**Fig. 3:**
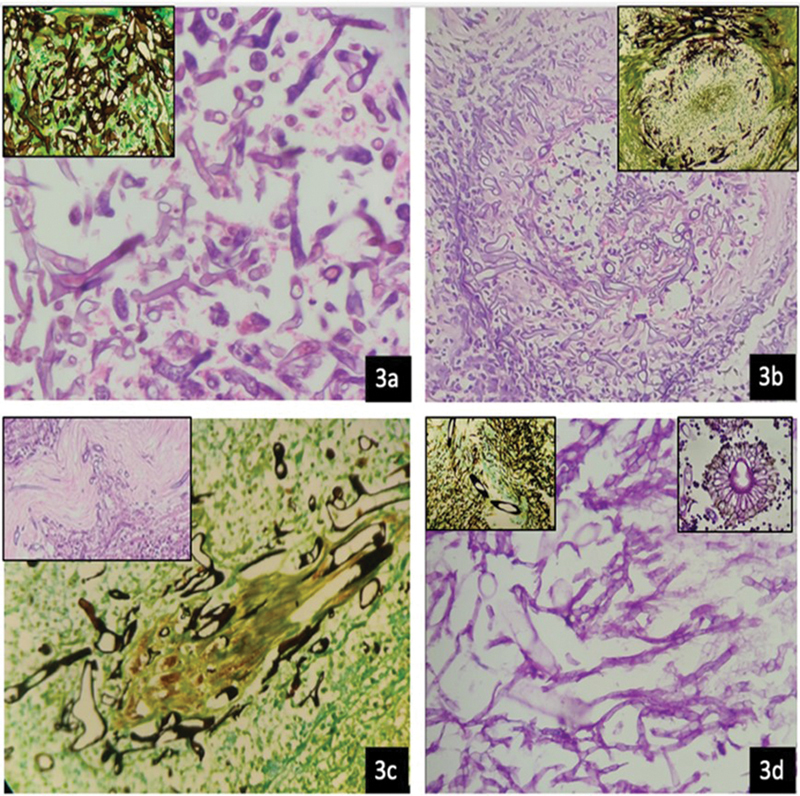
3a) H&E section from left maxillary sinus shows many, broad, aseptate hyphae with predominant 90 degree branching.(400x) Inset shows GMS stain. 3b) H&E section from right orbital tissue showing vessel wall invasion and luminal clogging by broad, aseptate hyphae (100x) Inset shows GMS stain of the same. 3c) GMS section from right orbital tissue showing neural invasion by fungi. (400x) Inset shows H&E stained section from orbital apex tissue with neural invasion by fungi. 3d) PAS stained section from nasal mucosa demonstrate presence of mixed fungi with broad aseptate hyphae (Mucor) and thin septate hyphae with an acute angle branching (Aspergillus)(400x). Inset (left) shows GMS stained section of the same and inset (right) shows fruiting body of Aspergillus.

**Fig. 4:**
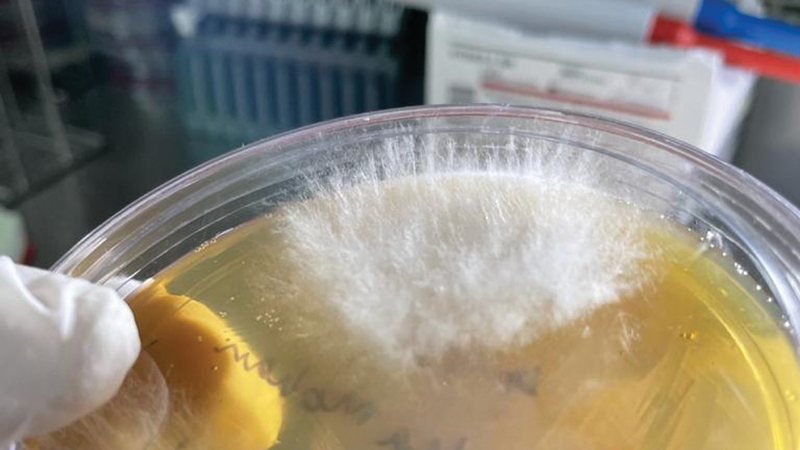
Sabarouds Dextrose Agar Plate showing growth of Mucor.

**Fig. 5:**
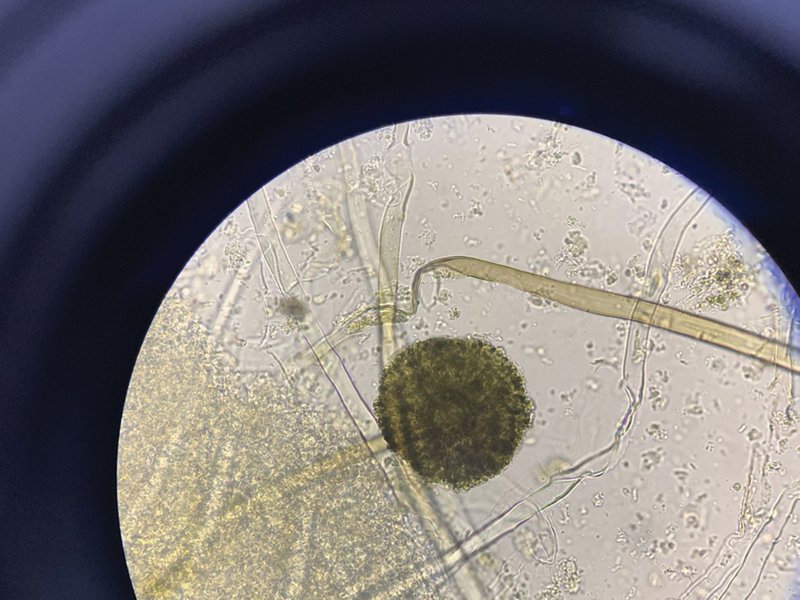
The Direct KOH Mount of Maxillary Sinus Tissue showing a mixture of thin septate, branching filamentous hyphae (suggestive of Aspergillus) and ribbon shaped, broad, aseptate hyphae (suggestive of Mucorales).

**Fig. 6:**
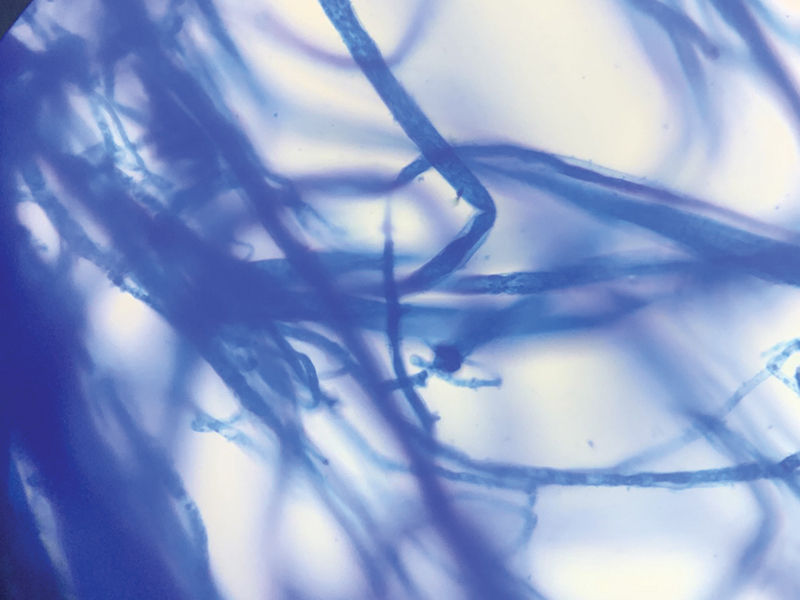
Lactophenol Cotton Blue Mount (LPCB) of Fungal growth showing broad, aseptate hyphae.

**Fig. 7:**
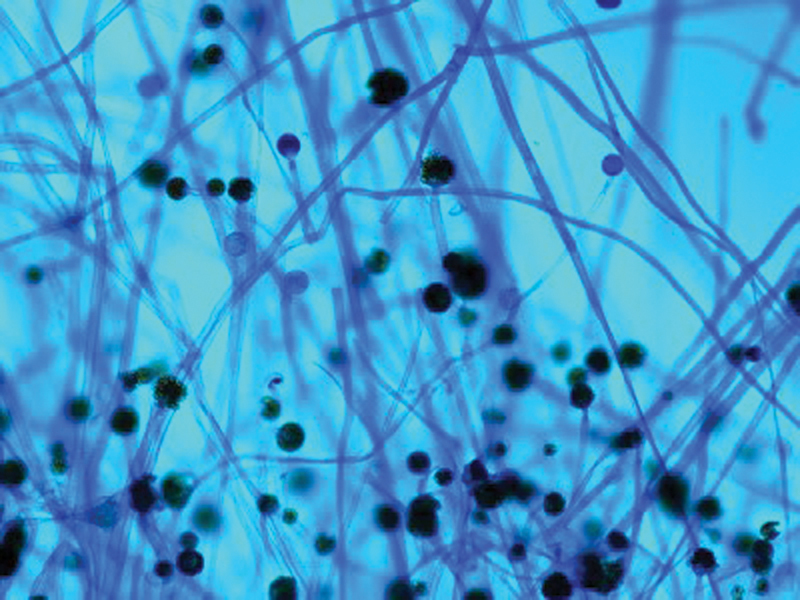
Lactophenol Cotton Blue Mount (LPCB) showing sparsely septated hyphae, sporangiospores bearing sporangia (suggestive of mucorales).

The samples received at microbiology laboratory were processed for bacterial and fungal culture. The samples were received in a sterile container with normal saline. The samples (tissues and fluids) were processed for potassium hydroxide (KOH) mount, gram stain, and cultured on Sabourauds dextrose agar ( Figs. 3 , 4 ). The tissue samples were cut into fine pieces, without crushing, and inoculated into Sabourauds dextrose agar with antibiotics (chloramphenicol, gentamicin), and in another tube without antibiotics. Each set of tubes was incubated at 37° C and 22° C, respectively. The samples were also inoculated in brain heart infusion broth and blood agar and incubated at 37° C. The cultures were viewed at 24 hours, 48 hours, 1 week, 2, 3, and 4 weeks for any fungal growth. The fungal isolates were finally identified by conventional techniques, like lactophenol cotton blue (LCB) mount, up to genus level. ( Figs. 5 , 6 , 7 )

**Type of Study –** Retrospective study.

**Study Period –** April 1, 2021, to June 25, 2021.

**Ethical Clearance –** The study was approved by Institutional Ethics Committee – AIG Hospitals.

**Statistical Analysis –** The data collected from the medical records was transferred into a separate study proforma. The data was entered into a Microsoft Excel spreadsheet (Microsoft Corp., Redmond, WA, USA) for further analysis after editing for completeness and consistency. The continuous variables were expressed as mean and standard deviation (SD), and categorical variables were expressed as % of frequency distribution. The Mann-Whitney U, *t* -, chi-squared, and Fisher exact tests were used. The analysis was performed by using the SPSS Statistics for Windows, version 20.0 software (IBM Corp., Armonk, NY, USA). *P* -value < 0.05 was considered as statistically significant.

## Results

The study was conducted from April 1, 2021, till June 25, 2021. One hundred twenty-one patients were included in the study. Two hundred fifty-two samples from different sites were collected from these patients. Out of 121 patients, 97 (80.2%) were males, 24 (19.8%) were females ( Table 1 ). The most common age group was > 50 years of age (49 males, 12 females), the next most common group was 41 to 50 years of age (28 males, 8 females). Out of 121 patients, 7 have died and 114 were discharged. Among the 97 male patients, 92 (94.8%) were alive and 5 (5.15%) had died, whereas among the females, 22 (91.66%) were alive and 2 had died (8.33%), the *p* -value was found to be significant ( *p* -value = 0.001).

**Table 1:** Total number of patients

	Alive	Dead
Males	92 (94.8%)	5 (5.15%)
Females	22 (91.66%)	2 (8.33%)

Out of the 121 patients, 88 were diagnosed with diabetes. Seventy out of these 88 patients (87.5%) were male, and 18/88 (20.45%) were female, and the *p* -value was found to be significant ( *p* -value = 0.001).

Eighty-eight out of 121 patients were treated with steroids, 77/88 males (87.5%) females are 11/88 (12.5%) were female, and the *p* -value was found to be significant ( *p* -value = 0.001).

The most common comorbidities seen in the patients were diabetes mellitus and hypertension.

**Fig. 8:**
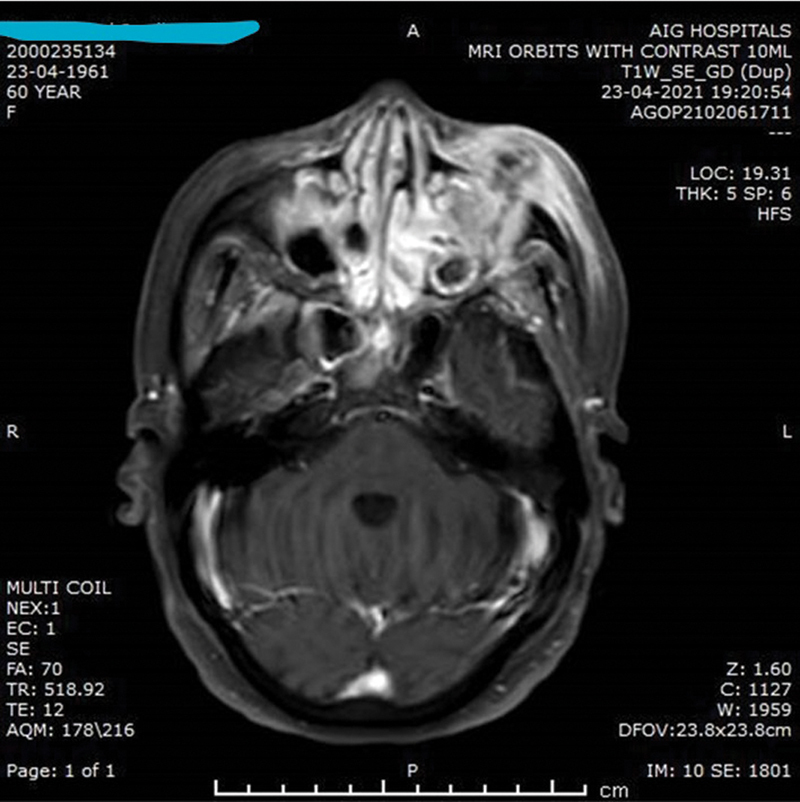
MRI OF PNS AND ORBITS Suspected face of Mucormycosis involving PNS and Left Orbit. Heterogenously enhancing contents in left maxillary sinus with extension into left orbit and soft tissues on left side of face. Similar heterogenously enhancing contents in bilateral ethmoidal air cells,bilateral sphenoid sinuses (R>L) and right maxillary sinus.

**Fig. 9:**
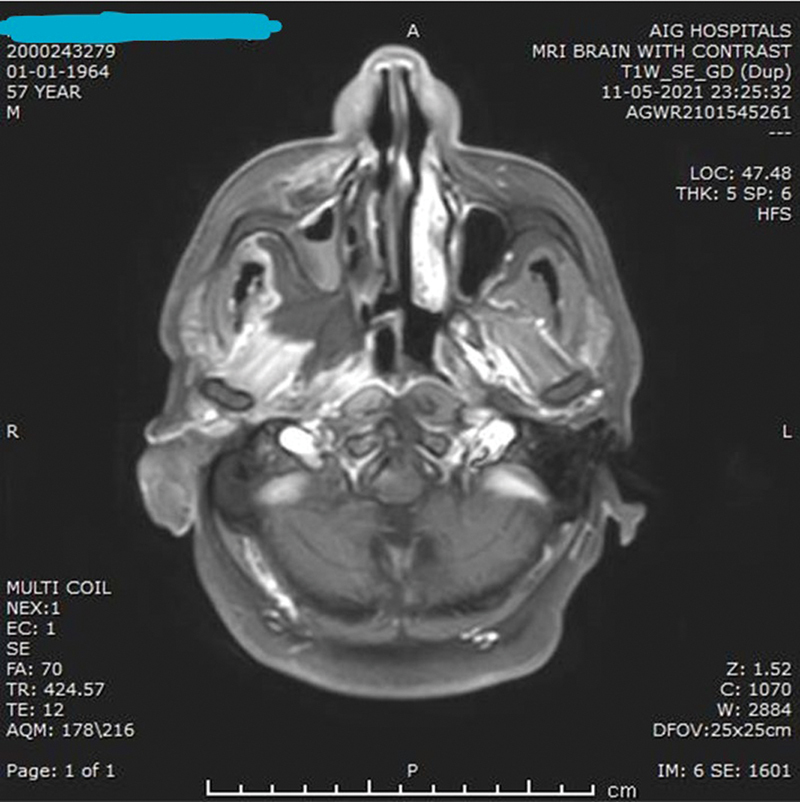
MRI OF BRAIN, PNS AND ORBITS 1. Sinusitis with necrosis involving the right maxillary, right ethmoid, bilateral frontal and sphenoid air cells. Extensive involvement of the right preantral, right retroantral soft tissue, the right masticator space, the right infratemporal fossa and the right palate with necrosis. Right-sided mastoiditis. 2. Changes of right-sided orbital and periorbital cellulitis with changes of optic neuritis,partially necrotic extraocular muscles with soft tissue in the right orbital apex and cavernous sinusFindings are in keeping with extensive rhio-orbital mucormycocis.

Among the 121 patients clinically diagnosed with fungal infections involving the sinuses, nose, and orbits, 95 (78.5%) had a positive laboratory diagnosis (either culture positive, KOH positive or positive histopathology report) ( Figs. 8 , 9 )

In 26 (21.5%) patients, all the tests were negative, even though they had clinical symptoms. Fungal culture was positive in 75 (61.9%) patients and histopathology report was positive in 62 (51.2%). Histopathology was positive in 7 (5.8%) patients whose culture and KOH tests were negative. ( Table 2 )

**Table 2:** Results of the 121 patients tested with different laboratory diagnostic tests

Culture result	KOH result	Histopathology result	Total nr. of patients
Negative	Negative	Positive	7
Positive	Positive	Positive	37
Negative	Positive	Negative	2
Negative	Negative	Negative	26
Positive	Negative	Positive	19
Negative	Positive	Positive	10
Positive	Negative	Positive	5
Positive	Positive	Negative	15

Out of 252 sites, 129 (51.1%) were collected from the maxilla, 69 (27.3%) from the ethmoid, 22 (8.73%) from the nose, 13 (5.15%) from the orbit, 10 (3.96%) from the sphenoid, 6 (2.38%) frontal, 2 (0.39%) from the lamina papyri mucosa, and 1 (0.39%) from the mastoid cells. ( Table 3 )

**Table 3:** Specimen collection sites

Name of the site	Total nr. of specimens collected
Maxilla	129 (51.1%)
Ethmoid	69 (27.3%)
Nose	22 (8.73%)
Orbit	13 (5.15%)
Sphenoid	10 (3.96%)
Frontal	6 (2.38%)
Lamina papyri mucosa	2 (0.79%)
Mastoid cells	1 (0.39%)
Total	252

Out of 116 fungal isolates obtained from 121 patients from 252 sites, ( Table 4 ), it was found that most of the fungal isolates belonged to Mucorales ( *Mucor* = 56, *Rhizopus* = 38, *Absidia* = 5) and rest being *Aspergillus* = 13 and *Alternaria* = 4.

**Table 4:** Fungal identification

Name of the fungus	Fungal culture positive	KOH	Histopathology	Site obtained from
Mucor N = 56	56	41-positive, 15-negative	36-positive, 7-negative, 5-chronic inflammation 8-acute inflammation	Maxilla-25 Ethmoid-23 Nose-7 Lamina payre-1
Rhizopus N = 38	38	31- positive 7-negative	23-positive for fungus 8- inflammation 7-negative	Maxilla-19 Ethmoid-12 Sphenoid-2 Orbit-2 Nose-2
Aspergillus N = 13	13	13-positive	3-positive for fungus 1- Acute inflammation 2-chronic inflammation	Maxilla-6 Nose-4 Orbit-1 Ethmoid-1 Sphenoid-1
Absidia N = 5	5	1-positive 4-negative	3-positive for fungal growth 1- Necrotic inflammation 1- Negative	Ethmoid-2 Middle turbinate (nose)-2 Frontal-1
Alternaria N = 4	4	4-negative	Acute inflammation-2 Chronic granulomatous inflammation-2	Ethmoid- 3 Left maxilla-1

Based on the distribution of the samples taken from 121 patients, it was observed that only 1 site was involved in 63 patients; 2 sites were involved in 40 patients; and 3 or more sites were involved in 18 patients ( Table 5 ). In 121 patients, the maxillary site was involved in 49 patients, the ethmoid site was involved in 6, and the remaining patients had combined involvement of the maxillary and ethmoid sites ( Table 6 ).

**Table 5:** Percentage distribution of the total number of samples taken from different sites among 121 patients

**No of sites**	**no**	**%**
one	63	52.1
two	40	33.1
three and above	18	14.8
total	121	100

**Table 6:** Percentage distribution of the type of sites

Name of site	no	%
Maxillary alone	49	40.5
Maxillary and combinations	55	45.5
Ethmoid one	6	5.0
Ethmoid and combinations	7	5.7
(Excluding maxillary)		
Others	4	3.3
Total	121	100

## Discussion

Coronavirus disease 2019 has been an eye opener, leaving the medical community with no clue about the exact treatment. Mankind had to bear the brunt of the destructive fungal infections which broke loose as a result of COVID-19 treatment. Especially with the Delta variant, hospitals were inundated with cases of COVID-19 survivors infected with various fungal species, which are actually thought to be innocuous unless the immune barriers have been breached. 17

Across the world, corticosteroids were one of the mainstay drugs administered for SARS-CoV-2 infected patients, as steroids would downregulate the SARS-CoV-2 replication and infectivity by modulating several cytokines, such as IL-4, 6, 8, 12, and tumor necrosis factor-α. 18 So SARS-CoV-2 and steroids which were used for the treatment, and also to attenuate cytokine release syndrome (CRS), both have led to immune modulation in affected patients, leaving them vulnerable to get infected by invasive fungal infections. 19 One of the major setback factors was prolonged duration of corticosteroids usage, which leads to fluid retention in the body, resulting in swelling, weight gain, and the creation of diabetes-like conditions. 20 These situations help the fungus to evade the host's immune system, and sometimes even a dormant fungal spore might bloom, increasing the chance of invasive fungal infections. ^5^ The study conducted by Leon-Borras et al. also revealed that usage of corticosteroids as treatment has led to 3.33 times increased risk of invasive fungal infections than in patients receiving nonsteroid treatment. 21

In addition to the ongoing agony, the lengthy intensive care unit (ICU) stay, requiring mechanical ventilation and various invasive devices, administration of steroids and antibiotics have made patients more prone to fungal infections. In addition to these factors, obesity has also, by and large, become one of the main predisposing factors as enlarged adipose tissues were apoptotic paving the way to immune cells attraction, causing inflammation due to the disorganized metabolic pathway of the fatty acid. 22

To date, various fungi like *Mucor* , *Aspergillus,* and *Candida* are being reported to cause invasive infections in COVID-19 survivors, causing infections due to immunity suppression caused either by steroids, diabetes, or due to an immune waned state created by covid infection.

Mucorales have predilection to invade blood vessels, causing thrombosis and dissemination leading to necrosis of the tissues commonly in cutaneous, pulmonary, gastrointestinal, rhino-orbital, or cerebral region. 23 The epithelial lining is damaged due to metabolic derangement such as diabetes, due to extensive medications or ventilation process. This leads to direct interaction of proteins with surrounding fungal spores, for instance, *Rhizopus* binds to collagen and laminin proteins using the endothelial glucose-regulator protein 78 (GRP78) receptor and endocytose itself inside the host and forms its hype, 24 leading to invasive fungal sepsis.

Diabetes and related ketoacidosis-like conditions lower the blood's pH, and high serum glucose, iron, acidic conditions, and β-hydroxyl butyrate, which impair the chelation of iron from transferrin, are all add-on factors that favor the growth of and invasion by fungus. Additionally, fungal contamination of medical supplies such as ostomy bags, humidifiers, nebulizers, suction canisters, linens, bandages can contribute to a fungal outbreak in hospital in COVID-19 patients. 25 Inhalation of these spores by patients during the hospital stay makes them susceptible hosts even after discharge from the hospital, and the spore viability depends on feasible conditions for their survival, such as higher iron levels in patient's serum. On the other hand, patients with no known risk factors were also registered, implying that the virus and the medications administered could also cause immune suppression, exposing patients to opportunistic fungi. 24

Singh et al. have highlighted that the prevalence rate of mucormycosis was ∼ 81.2% in India when compared with the whole world, with a mortality rate of 30.7%. 26 Whereas in our study, the prevalence rate of fungal infections was 78.5%, and the mortality rate was 5.7%

*Aspergillus* produces conidiospores, which are engulfed by alveolar macrophages, triggers proinflammatory reaction, and recruits neutrophils at the site of infectivity in immunocompromised patients. They evade the macrophages, germinate, and invade in the lumen of blood vessles. They block pathogen-associated molecular patterns (PAMPs), increase catalase production, mannitol, and superoxide dismutase, and increase the production of secondary metabolic products like fumagillin, cytochalasin E, gliotoxin, and actibind. A peculiar feature of *Aspergillus* is producing melanin pigments to protect its spores from environmental conditions; this helps in scavenging the reactive oxygen intermediates in host cells masking β-glucans, trafficking conidiospores intracellularly, thus causing infection. 27

Zuo et al. have shown that the microbiota of COVID-19 patients is altered, due to reduced T-cell production in the host's body. 28

Diao et al. have shown the presence of the *Candida* and *Aspergillus* species in the stool of COVID-19 patients, which might trigger secondary infections in the postrecovery phase of COVID-19 survivors, leading to alteration in the respiratory and intestinal mycobiomes. 29

From our study, we have made an observation that specially to detect fungal presence, subjecting a sample to different laboratory tests has always been high yielding. Though all the patients who were considered in our study were clinically symptomatic, 26 had not shown anything positive in the different laboratory tests done (histopathology, KOH mount, and fungal culture). This could be due to the hyphal elements of the fungus, especially mucormycetes, being damaged due to excessive grinding or sectioning of the tissue.

In cases in which all the modalities are negative, immunodiagnostics, molecular techniques, and immunochemical staining methods (which we have not done in our study) can be useful in diagnosing fungal infections, especially mucormycosis. Frozen section evaluation is also an important modality to provide fast and valuable information about the pathology involved.

In the rest of the samples, one of the laboratory tests was positive. Though KOH was positive, not all KOH-positive samples have shown culture or histopathology positivity. This may be due to sparse distribution of the fungus in the infected tissue and missing out or breakage of slender hyphae while processing the specimen. And the drawback can be that different bits of sample are used for different tests, and there may be a variability in the presence of the fungus. However, in samples which did not show fungus on histopathology, neutrophilic inflammatory response was observed. 30

## Conclusion

In COVID-19 patients treatment methods, administration of immune suppressants, and antibiotics, with an intention to salvage, have made patients susceptible to benign fungi, causing them to evade the host's immunity, thus leading to invasive infections.

In samples received from patients who were clinically and radiologically diagnosed with fungal infection, a positive laboratory diagnosis would confirm the clinical suspicion and help in planning the course of treatment; for example, a quick microscopy result while the patient was still in the operation theater, in our setting, has also helped the surgeon in deciding the extent of debridement to be done. Therefore, applying different laboratory modalities would not only aid in providing fast and valuable information but also in understanding the pathology and assisting the clinician in selecting the correct treatment for the patient.
